# Chitosan Hydrogel Beads Functionalized with Thymol-Loaded Solid Lipid–Polymer Hybrid Nanoparticles

**DOI:** 10.3390/ijms19103112

**Published:** 2018-10-11

**Authors:** Taoran Wang, Yangchao Luo

**Affiliations:** Department of Nutritional Sciences, University of Connecticut, Storrs, CT 06269, USA; taoran.wang@uconn.edu

**Keywords:** chitosan hydrogel beads, functionalization, nanoparticles, thymol, encapsulation

## Abstract

In this study, the innovative and multifunctional nanoparticles–hydrogel nanocomposites made with chitosan hydrogel beads and solid lipid–polymer hybrid nanoparticles (SLPN) were prepared through conjugation between SLPN and chitosan beads. The SLPNs were first fabricated via coating the bovine serum albumin (BSA)-emulsified solid lipid nanoparticles with oxidized dextran. The aldehyde groups of the oxidized dextran on the surface of the SLPN enabled an in situ conjugation with the chitosan beads through the Schiff base linkage. The obtained nano-on-beads composite exhibited a spherical shape with a homogeneous size distribution. The successful conjugation of SLPN on the chitosan beads was confirmed by a Fourier transform infrared spectroscopy and a scanning electron microscope. The effects of the beads dosage (50, 100, 200, and 300 beads) and the incubation duration (30, 60, 90, 120, and 150 min) on the conjugation efficiency of SLPN onto the beads were comprehensively optimized. The optimal formulations were found to be a 200 bead dosage, with 30–90 min incubation duration groups. The optimal formulations were then used to encapsulate thymol, an antibacterial agent, which was studied as a model compound. After encapsulation, the thymol exhibited sustained release profiles in the phosphate buffer saline. The as-prepared nanoparticles–hydrogel nanocomposites reported in this proof-of-concept study hold promising features as a controlled-release antibacterial approach for improving food safety.

## 1. Introduction

Hydrogels are highly hydrated three-dimensional network structures that are able to absorb large quantities of water and can swell without being dissolved, as a result of chemical or physical cross-linking [1]. Because of their high water content, as well as their physiochemical similarity to the native extracellular matrix, hydrogels are extensively employed in various applications such as tissue engineering, bio-adsorbents, and drug delivery systems [2,3,4]. Depending on the techniques involved in the preparation process, the appearance of hydrogels could be matrix, film, microsphere, or beads [1]. Hydrogels could be prepared via gelation using the mechanism of antisolvent coagulation; ionic or covalent cross-linking of hydrophilic synthetic polymers, such as polyethylene glycol (PEG), polyacrylamide (PAM), and polyvinyl alcohol (PVA); or natural polysaccharides, such as alginate and chitosan. However, some synthetic polymers such as PAM and its derivatives, acrylamide (AM) and ethyleneimine, are extremely toxic and can cause severe neurotoxic effects [5,6]. Moreover, many widely used cross-linkers, such as glutaraldehyde, are known to generate potential cytotoxicity, which could compromise further applications of hydrogels cross-linked by these linkers [7]. Therefore, hydrogels prepared using biopolymers that have been prepared with safe and effective cross-linking methods, have attracted increased attention in recent years. Among the biopolymer-based hydrogels, chitosan hydrogel beads have been extensively applied in various fields, because of the remarkable properties of chitosan, such as its low toxicity, biodegradability, and biocompatibility [8,9].

Currently, the functionalization of chitosan hydrogel has become more and more popular, as it could confer additional functions to native chitosan hydrogel. In particular, nanoparticles–chitosan hydrogel composites have been developed and studied over decades, because of their enhanced properties compared with their individual components [10]. For instance, the recently reported metal nanoparticles–chitosan hydrogel composite made of silver or gold nanoparticles and chitosan has demonstrated an exceptional antimicrobial activity and an adsorption ability of heavy metals or pesticides [11,12,13]. Furthermore, silver nanoparticles–functionalized chitosan hydrogels exhibited no negative impact on the cells of dermis compared with individual silver nanoparticles [11,14,15]. However, the release and migration of metal nanoparticles from the hydrogel matrix to the environment may pose serious safety concerns by introducing secondary contamination, which may cause severe adverse consequences to human health and the natural environment [16,17]. Therefore, developing novel methodologies to functionalize chitosan-based hydrogels for the preparation of nanocomposites is critically needed. In this study, we have proposed preparing a novel nano-on-beads composite using chitosan hydrogel and organic nanoparticles (i.e., solid lipid–polymer hybrid nanoparticles (SLPN)). The nanoparticles were crosslinked onto chitosan hydrogel beads via food-grade macromolecular crosslinkers to create the covalent bonding, thus minimizing the migration of the nanoparticles from the beads.

In our previous study, we successfully prepared SLPN from oxidized dextran (OD), bovine serum albumin (BSA), and solid lipid [18]. OD is a dialdehyde polymer obtained from the oxidation of native dextran. The abundant aldehyde groups on OD not only enable an in-situ crosslinking with amino groups on BSA during the preparation of SLPN, but also makes SLPN capable of reacting with other amino-containing polymers via the Schiff base linkage. The chitosan hydrogel beads, prepared using a coagulation technique, acted as a polymeric hydrogel matrix rich in amino groups for reacting with SLPN. Thus, we proposed that the OD-decorated SLPN could be covalently conjugated to the surface of the chitosan beads to form nano-on-beads composite, by simply incubating the beads in a colloidal SLPN dispersion. The major objective of this study is to prove this innovative concept and to prepare and comprehensively characterize the obtained nano-on-beads composite, including its size, composition, and morphology.

## 2. Results and Discussion

### 2.1. Functionalization of Chitosan Beads with SLPN

**Figure 1:**
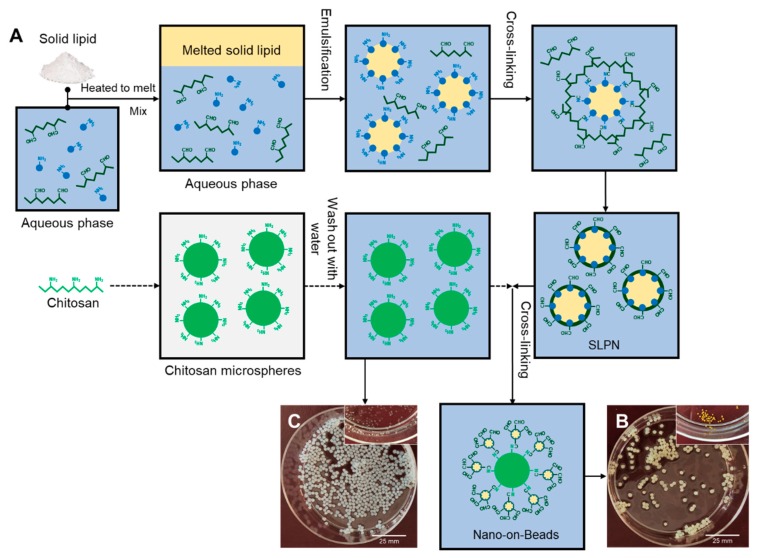
(**A**) The formulation of solid lipid–polymer hybrid nanoparticles (SLPN) and the fabrication of nano-on-beads composite with as-prepared SLPN; (**B**) hydrated and dehydrated (top-right) states of SLPN functionalized chitosan beads; (**C**) hydrated and dehydrated (top-right) states of pure chitosan beads.

In our previous study [18], oxidized dextran (OD) was successfully prepared by the oxidation of native dextran with sodium periodate (NaIO_4_). The structure of OD was confirmed by nuclear magnetic resonance (NMR) spectroscopy and Fourier-transform infrared spectroscopy (FTIR). The prepared OD was then used in the formulation of SLPN by cross-linking it with BSA in order to confer the aldehyde groups on the surface of the nanoparticles, resulting in the formation of surface-active SLPNs capable of reacting with other amino-containing polymers, such as chitosan. Therefore, in the present study, we first prepared the chitosan beads using an antisolvent coagulation method, using a concentrated sodium hydroxide (NaOH) solution, and then these beads were subsequently incubated with surface-active SLPN for functionalization via the Schiff base linkage (Figure 1A). For comparison, chitosan beads with and without SLPN were both prepared, and their digital photos are shown in Figure 1B,C, respectively. Apparently, both types of beads had a spherical shape with a homogeneous size distribution, while the beads functionalized with SLPN exhibited a slightly larger dimension (2–2.5 mm) than the control beads without functionalization (2.2–2.7 mm). In the hydrated state, the pure chitosan beads appeared to be white, while the color of the SLPN-chitosan nanocomposite beads varied from light yellow to orange, depending on the heating duration, due to the extent of the Schiff base formation. Upon dehydration, a significant shrinkage was observed for both types of beads, as the water molecules diffused out. In the dried state, the pure chitosan beads changed into hemispherical shape, whereas the SLPN-chitosan–nanocomposite beads were able to maintain their original spherical shape without a noticeable collapse on the surface, indicating an improved mechanical property after functionalization [19]. The enhanced strength of the SLPN-chitosan beads could be attributed to the conjugation of SLPN on the surface, as well as the crosslinking effect of the free OD present in the SLPN dispersion. It has been previously reported that the dialdehyde biomacromolecules are very effective crosslinkers to chitosan for forming chitosan hydrogels [20,21,22].

### 2.2. Effect of Chitosan Beads Dosage and Incubation Duration

**Figure 2:**
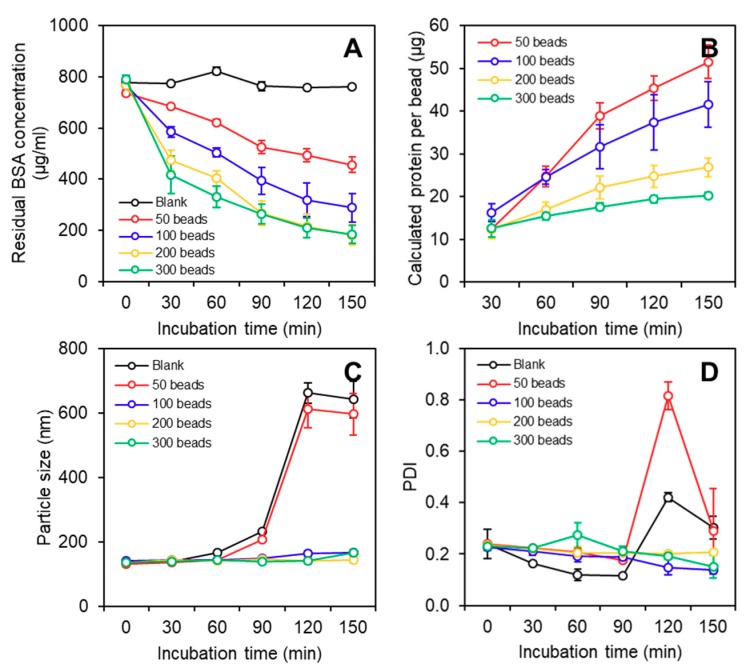
(**A**) Change of protein concentration in the SLPN dispersion during incubation with chitosan beads; (**B**) calculated protein amount per bead during the incubation of SLPN with chitosan beads; particle size (**C**) and polydispersity index (PDI) (**D**) of the SLPN dispersion during incubation with chitosan beads.

To investigate the effects of the beads dosage (50, 100, 200, and 300 beads) and incubation duration (30, 60, 90, 120, and 150 min) on the conjugation efficiency of SLPN onto the chitosan beads, the protein concentration in the SLPN dispersion was measured at different time points during the functionalization process. The protein, BSA, was used as a natural emulsifier during the SLPN preparation, and it formed the intermediate layer between the OD coating and the solid lipid core. While there may be free OD in the SLPN dispersion, the BSA molecules, as the intermediate and amphiphilic layer in the SLPN structure, shall be adsorbed on the solid lipid core. Thus, the change in the BSA concentration in the SLPN dispersion during the conjugation process was correlated to the change of the SLPN concentration. By subtracting the measured residual BSA concentration in the dispersion from the total BSA concentration used to prepare the SLPN, one can deduce the SLPN conjugated to the chitosan beads and thus calculate the conjugation rate and efficiency. Figure 2A shows the change of the residual BSA content in the SLPN dispersion as a function of the incubation time and the number of beads. Generally, more SLPNs were conjugated to the surface of the chitosan beads over time for a given number of beads, and the conjugation rate was greater in the groups with a larger number of beads. In particular, in the groups of 200 and 300 beads, a burst reduction of BSA concentration was observed within the first 30 min, being 50% and 35%, respectively, followed by a linear reduction. In contrast, in the groups of 50 and 100 beads, such a burst reduction was not noticeable. This could be due to the increase in the number of absorption sites (amino groups on chitosan beads) when more chitosan beads were present.

Moreover, the protein content was normalized based on the number of beads, and the calculated protein concentration per bead was higher at lower bead dosage group (Figure 2B), suggesting that more SLPNs were conjugated to one bead when a smaller number of beads were present. Although incubation with less beads in a given amount of SLPNs could achieve a higher concentration of nanoparticles per bead, the physicochemical characteristics of the SLPN dispersion during the conjugation process needs to be considered, as the extended heating time is involved, which may alter the nanoparticle structure of the SLPNs. During the incubation and crosslinking process, not only were the SLPNs covalently bonded onto the surface of the chitosan beads, but the free OD in the dispersion also reacted with the SLPN and chitosan hydrogel matrix. Our previous study demonstrated that with the increased heating time, the formation of significantly larger SLPNs and even the aggregation and precipitation of SLPNs occurred [18]. The reaction between the free OD and the BSA in the SLPN led to a change in the particulate characteristics of the SLPN, probably due to the excessive coating. In this study, the characteristics of the SLPNs were monitored by measuring the particle size and polydispersity index (PDI) of the SLPNs in the dispersion, throughout the incubation time during conjugation. Before crosslinking (time 0 min in Figure 2C,D), the particle size and PDI of the prepared SLPN was around 137 nm and 0.23, respectively. During the conjugation process, the SLPNs in all of the groups were able to maintain their original particle size and PDI in the first 60 min, while the particle size of the blank and 50-beads groups started to increase slowly to over 200 nm at 90 min, and then rapidly to over 600 nm at 120 min. Concomitantly, following a similar trend, the PDI of these two groups also significantly increased to 0.4 and 0.8, respectively. While the dramatic increase in the particle size in the blank group that did not have any chitosan beads was well corroborated with our previous study, where excessive heating induced the SLPN aggregation [18]; the different stability of SLPN in the groups with a varying number of beads may be explained, as below. As OD has no distance restriction to promote cross-linking, it could rapidly cover the surface of the BSA molecules during the SLPN preparation rather than forming intermolecular conjugation among the protein molecules like conventional cross-linkers (e.g., glutaraldehyde) do [23]. It must be noted that the free ODs that did not react with BSA during the preparation of SLPN were not removed from the dispersion, and therefore the free ODs would continue to react with the already-formed SLPN in the heating process during the preparation of the nano-on-beads composite, leading to an excessively thick OD layer depositing on the SLPN and a significantly larger particle size with a greater PDI. Interestingly, for the other groups with a greater number of beads (i.e., 100, 200, and 300 beads), the particle size and PDI remained constant throughout the conjugation process, up to 150 min. The differences in the particulate characteristics of the SLPNs among the different groups during incubation could be attributed to the fast uptake and clearance of free OD by the superfluous beads in the groups with more than 100 beads. In particular, not only did the amino groups of BSA in SLPN react with the free OD, but the amino groups from the chitosan hydrogel matrix reacted as well. Therefore, when there were enough beads that could help to quickly react with free the OD in the dispersion, the free OD were spared from reacting with the SLPN, preventing the formation of an unnecessarily thick coating, and thus improving the stability of the SLPNs against aggregation during functionalization. In other words, the reaction rate between the free OD with 50 chitosan beads was not fast enough to competitively inhibit or slow down the reaction between the free OD and SLPN, resulting in SLPNs that failed to maintain their particle size and PDI after a long incubation period. As a result, the chitosan beads in the 50-beads group may be functionalized by the aggregated SLPNs. Collectively, based on the obtained results, the groups with 200 beads at three time points (i.e., 30, 60, and 90 min) were selected for the following studies.

### 2.3. Characterization of Composite Beads

**Figure 3:**
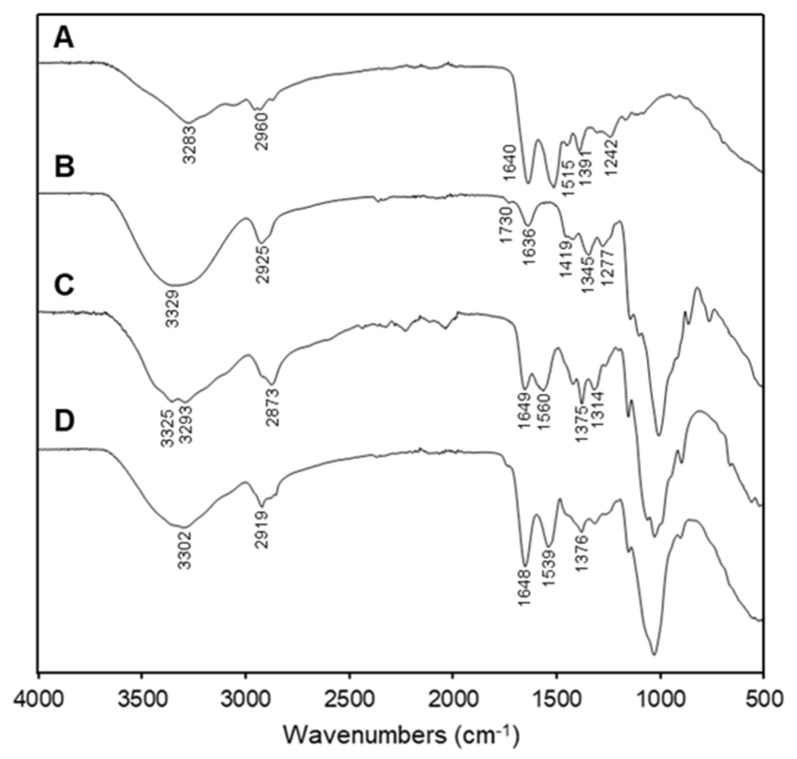
The FTIR spectra of (**A**) bovine serum albumin (BSA), (**B**) oxidized dextran (OD), (**C**) chitosan beads, and (**D**) 200B-90m bead.

**Figure 4:**
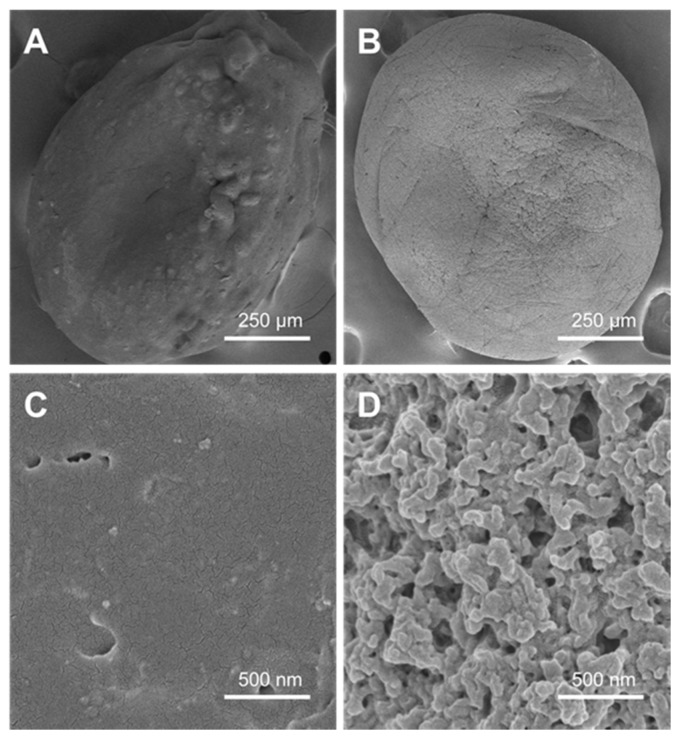
SEM images of (**A**) an oven dried chitosan bead, (**B**) oven dried nano-on-bead composite (200B-90m), (**C**) oven dried chitosan bead showing surface detail, and (**D**) oven dried nano-on-bead composite (200B-90m) showing surface detail.

Figure 3 shows the FTIR spectra of the individual components, of the SLPN and the selected nano-on-beads composite sample. In the spectrum of the native BSA, two major characteristic peaks at 1640 and 1515 cm^−1^ were detected and assigned to the amide I and amide II stretching vibrations, respectively (Figure 3A). The OD spectrum exhibited some typical characteristics of an absorption band of native dextran, including 3329 cm^−1^ due to O–H stretching; 1636 cm^−1^ due to water molecule bending; and 2925, 1419, and 1345 cm^–1^ assigned to ν(C–H) and δ(C–H) vibrational modes [24]. Furthermore, the dialdehyde absorption peak at 1730 cm^–1^ was observed in the OD spectrum (Figure 3B). The spectrum from the pure chitosan beads showed signals at 1649, 1560, and 1314 cm^−1^ for the C=O stretching (amide I), N–H bending (amine II), and C–N stretching (amide III), respectively [25,26]. The chitosan spectrum also exhibited some characteristic peaks of polysaccharide, such as O–H stretching, C–H stretching, and C–O stretching at 3400–3600, 2800–2900, and 1020–1180 cm^−1^, respectively (Figure 3C) [27]. After functionalization with SLPN, the N–H stretching at 3325 cm^−1^ and the N–H bending at 1560 cm^−1^, which were originally present in the pure chitosan beads, could not be detected in the composite beads (Figure 3D). Based on a previous study, the Schiff bases were found to exhibit an absorption band at 1613–1631 cm^−1^ [28]. However, it is hard to identify this peak in the spectra of the composite beads, which might be due to the overlay of the imine bond with the amide bond from both BSA and chitosan. There were no significant differences between the nano-on-beads groups (data now shown). Furthermore, the surface morphology of the selected oven-dried beads was visualized by SEM (Figure 4). The chitosan bead without functionalization had rough surface with diameter around 750 µm (Figure 4A,C), which was concordant with other studies on chitosan beads prepared using the coagulation technique [29,30]. After functionalization with SLPN, although no change in the size of the dried beads, the clusters of nanoparticles with size ranging from 100 to 200 nm were clearly observed on the surface of the chitosan bead (Figure 4B,D), demonstrating the successful conjugation of SLPN on the bead.

In addition to confirming the functionalization of beads with SLPN, the detachment or migration behavior of SLPN from the chitosan beads when incubated in a buffer medium was then evaluated by measuring the time-dependent protein concentration in the supernatant. Interestingly, the BSA concentration was not be detected in the supernatant at all within the investigated time period, up to 24 h. This result suggested that covalent bonding through the Schiff base linkage between the chitosan and SLPN was strong enough to retain an SLPN from the migration into the buffer medium.

### 2.4. Encapsulation of Thymol and Functionalization

**Figure 5:**
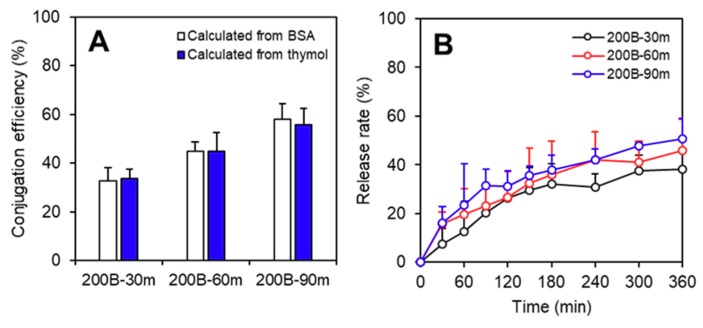
(**A**) Conjugation efficiency of SLPN on chitosan beads from different calculation methods; (**B**) release profile of thymol from nanoparticles.

SLPNs with thymol loading at 0.05, 0.075, and 0.1 mg/mg (*w*/*w*, thymol/lipid) were successfully fabricated and were denoted as T-SLPN1, T-SLPN2, and T-SLPN3, respectively. The particulate characteristics and encapsulation efficiency are shown in Table 1. Compared to the empty SLPN, the particle size, PDI, and absolute value of the zeta potential slightly increased with the increase of the thymol loading. When the concentration of thymol reached 0.1 mg/mL, the encapsulation efficiency of SLPN significantly decreased from around 92% to 78%, indicating that SLPN may not be capable of accommodating such a high concentration of thymol. Thus, 0.075 mg/mL of loading SLPNs were selected for further experiments. Subsequently, the thymol-loaded SLPN (T-SLPN2) was used to functionalize the chitosan beads following the protocol established in this study (i.e., 200B-30m, 200B-60m, and 200B-90m). As thymol is a hydrophobic molecule that is supposed to be entrapped in the core of SLPN, the residual concentration of thymol in the T-SLPN2 dispersion can be used as another indicator, in addition to BSA, for calculating the conjugation efficiency. Therefore, during the functionalization of the chitosan with T-SLPN2, the thymol concentration was measured using UV-VIS spectroscopy, in order to quantitatively confirm and validate the absorption of SLPN onto beads. As shown in Figure 5A, the conjugation efficiencies calculated from the measurement of the residual thymol concentration in the T-SLPN dispersion after functionalization were well comparable with the data calculated BSA concentration. The results again demonstrated the successful functionalization of chitosan beads by SLPN via an in situ cross-linking reaction.

The cumulative release of thymol from the composite beads in the phosphate buffered saline (PBS) buffer is presented in Figure 5B. Three formulations of composite beads all exhibited controlled release profiles, with less than 50% thymol detected in the release medium after 6 h of incubation. Several mathematical models including the zero-order, Higuchi, and Ritger–Peppas models were applied based on previous studies, in order to understand and elucidate the release mechanism of thymol from the nano-on-beads composite system [31,32]. The model simulation and data analysis were done by using R and RStudio software, and the results are shown in Table 2. Generally speaking, the kinetic release mechanism of thymol was best explained by the Higuchi model, which had the highest determination of coefficient (R^2^). The Higuchi model explained that thymol was first to be dissolved in a solid lipid matrix and was diffused to the surface of the matrix, then partitioned to amphiphilic protein layer, followed by the hydrophilic OD layer. Finally, the thymol was released to the surrounding medium. Among all of the groups, the 200B-30m group presented the highest R^2^ in the zero-order model (ideal controlled release kinetic with constant release rate at all time), which means that the release of thymol was a better control in the 200B-30m group than in the other groups. As we had demonstrated that SLPNs maintained their integrity, and that the detachment of BSA from the composite beads was not detected for over 24 h of incubation in the same PBS medium, one could conclude that the sustained release of thymol from the composite beads was governed by diffusion.

Thymol has been proven to have potent antimicrobial activities against a wide spectrum of pathogenic pathogens found in food products, including *L. monocytogenes*, *S. typhimurium*, *E. coli* O157:H7, and *B. thermosphacta* [33,34,35,36]. The minimum inhibitory concentration (MIC) and minimum bactericidal concentration (MBC) values for *L. monocytogenes* and *S. typhimurium* ranged from 0.25 to 0.5 μL/mL (0.24 mg to 0.48 mg/mL), while *E. coli* O157:H7 was less inhibited by thymol, with MIC and MBC ranging from 0.5 to 1 μL/mL (0.48 mg to 0.96 mg/mL). In our study, the thymol amount per bead (T-SLPN2) was 0.0013, 0.0017, and 0.0021 mg for 200B-30m, 200B-60m, and 200B-90m, respectively. The nanoencapsulation of thymol in SLPN is expected to enhance its antimicrobial activity by providing a large surface area, and it thus has better contact with the bacteria. By developing the nano-on-beads composite, thymol-loaded nanoparticles are immobilized on the chitosan beads, and when an appropriate number (e.g., 100–400 beads) of composite beads is used, it will ensure that the concentration of thymol reaches its MIC and MBC values against different bacteria, enabling the antimicrobial potency against common pathogenic pathogens.

## 3. Materials and Methods

### 3.1. Materials

Precirol^®^ ATO 5 was a kind gift from Gattefossé. Dextran (40 kDa), thymol, sodium periodate (NaIO_4_), bovine serum albumin (BSA), and low molecular chitosan (75–85% deacetylated) were purchased from Sigma-Aldrich (St. Louis, MO, USA). Hydrochloric acid (HCl), sodium hydroxide (NaOH), and ethanol were obtained from Fisher Scientific Co. (Norcross, GA, USA). Unless noted otherwise, all of the chemicals were of analytical grade and were used without further purification.

### 3.2. Preparation of Chitosan Hydrogel Beads

The chitosan solution (2% *w*/*v*) was prepared by dissolving chitosan in 1% (*v*/*v*) acetic acid at room temperature overnight, with gentle stirring. In order to eliminate the gas bubbles, the chitosan solution was sonicated for 3 min, and then gently stirred overnight for complete hydration. To prepare the chitosan beads, 5 mL of the chitosan solution was extruded dropwise into a beaker containing 25 mL of 1 M sodium hydroxide, through tubing connected to a 200 µL pipette tip, and the extrusion was powered by a Fisherbrand™ FH30 peristaltic pump (Norcross, GA, USA). The obtained chitosan beads were solidified in sodium hydroxide for 2 h while stirring at 300 rpm. Then, the alkaline solution was slowly decanted and the chitosan beads were collected using filtration. The beads were successively washed with ultrapure water, until the eluted water was neutral.

### 3.3. Preparation of SLPN

Before the preparation of SLPN, the OD was synthesized based on previous literature [37]. Dextran (40 kDa) was used to prepare the OD, and the entire oxidation procedure was performed in a beaker protected from light. Briefly, the dextran solution (2.4 g/50 mL) was reacted with 0.2 M sodium metaperiodate (50 mL) for 24 h at room temperature at pH 4. After the reaction, the mixture was dialyzed against water for 24 h, followed by lyophilization. The obtained OD powder was stored in 4 °C until use. The oxidation degree was determined to be 28.1%, as reported in our recent study [18].

Empty SLPNs were prepared using a homogenization and sonication method, as previously reported [38]. Briefly, 10 mg of Precirol^®^ ATO 5 powder was heated to melt at 65 °C. Then, 10 mL of pre-heated (65 °C) aqueous phase containing 1 mg/mL BSA and 1 mg/mL OD solution in pure water was added into the above melted lipid under homogenization at 25,000 rpm for 30 s, to obtain a coarse emulsion, followed by 3 min sonication using a probe-type sonicator (Misonix Sonicator^®^ 3000, Vernon Hills, IL, USA). The obtained sample was incubated under 70 °C for 30 min to initiate the in situ conjugation between the BSA and OD. Subsequently, the mixture was rapidly cooled down in an ice bath to solidify the solid lipid core.

### 3.4. Encapsulation of Thymol in SLPN

With the attempt to prepare antimicrobial hydrogel nanocomposite, thymol, an essential oil with a potent antimicrobial activity, was loaded into SLPN. Briefly, thymol was dissolved in ethanol at a concentration of 10 mg/mL as a stock solution. Then, different amounts of thymol stock solution (50, 75 or 100 μL, equivalent to 0.5, 0.75, and 1 mg thymol, respectively) were mixed with the melted solid lipid to form new lipid phases, and were incubated together at 70 °C for 1 min. The subsequent procedures were the same as described above for the preparation of the empty SLPNs.

The concentration of the thymol encapsulated in the SLPNs was measured using UV-VIS spectroscopy. The encapsulation efficiency (EE) was determined by measuring the concentration of the un-encapsulated thymol, using an Amicon^®^ Ultra centrifugation device with a molecular weight cutoff of 10 kDa. After centrifugation under 10,000× *g* for 15 min, the solution in the receiving reservoir was collected and diluted five times with hexane, so as to extract the free thymol. The concentration of the extracted thymol was measured using a UV-VIS spectrophotometer at 275 nm, with a pre-established thymol standard curve (0.01–0.1 mg/mL). The EE was calculated using the following equation:

$$\mathrm{EE}\;\left(\%\right)=\frac{\mathrm{Total}\;\mathrm{thymol}-\mathrm{filtered}\;\mathrm{thymol}}{\mathrm{Total}\;\mathrm{thymol}}\times 100\%$$

### 3.5. Functionalization of Chitosan Beads with SLPN

A certain dosage of chitosan hydrogel beads (50, 100, 200, and 300 beads) were directly added into a solution of freshly prepared SLPN solution (with or without thymol loading). The mixture was incubated under 70 °C for 30, 60, 90, 120, and 150 min so as to initiate the functionalization via a Schiff base reaction between the amino groups of the chitosan and aldehyde groups of the OD. After incubation, the mixture was cooled down in an ice bath. To determine the adsorption efficiency of SLPN onto the chitosan beads, the concentration of protein in the supernatant was determined at designated time points (30, 60, 90, 120, and 150 min) using a protein assay kit, according to the manufacturer′s protocol. To determine the particulate characteristics of the SLPNs after functionalization, the particle size, PDI, and zeta potential of the SLPNs were measured using a Zetasizer Nano ZS (Malvern Instruments Ltd., Worcestershire, UK). The particle size was determined by dynamic light scattering (DLS) at a 173° scattering angle. The PDI, which is a parameter to evaluate the homogeneity of the nanoparticles, was recorded together with particle size measurement. The zeta potential was calculated from the electrophoretic mobility of the sample. The SLPN functionalized chitosan beads are hereafter denoted based on their beads amount and incubation time. For example, the 50 chitosan beads with 30 min incubation group was labelled as 50B-30m.

### 3.6. Characterization of SLPN Functionalized Chitosan Beads (Nano-on-Beads Composite)

Selected samples (200B-30m, -60m, and -90m) were placed in aluminum foil plate and dried in the oven for 2 h at 40 °C. The preliminary data indicated that drying at 40 °C for 2 h was sufficient to completely remove any water from the beads by measuring the weight change as a function of the drying time. After drying, the Fourier-transform infrared spectroscopy (FTIR) spectra of dried beads were recorded using a NicoletTM iSTM5 FT-IR spectrophotometer (Thermo Scientific, Waltham, MA, USA). in the range of 500–4000 cm^−1^. The results were analyzed using OMNIC software, version 8.0.

The oven-dried bead samples were directly placed on double-sided carbon tape. All of the samples were coated with gold using a sputter coater, before being observed under a scanning electron microscope (SEM, JSM-6335F, JEOL Ltd., Tokyo, Japan).

To evaluate the detachment of the SLPN from the chitosan beads, samples were incubated in water under mild stirring, and the incubation medium was collected at designated time points (1, 3, 6, 12, and 24 h) for the measurement of the protein concentration using a Lowry assay, according to the manufacturer′s protocol.

### 3.7. Release Study

The release rate of the thymol from the nano-on-beads composite was conducted in phosphate buffered saline (PBS, 10 mM). The selected thymol loaded nano-on-beads composite (200 beads) were directly placed in a PBS release medium (50 mL). The release system was carefully closed so as to prevent evaporation during the release test. During the experiment, 2 mL of the release medium was withdrawn at predetermined time intervals, with the replacement of the same volume of fresh medium in order to keep the volume constant. The collected release medium was then lyophilized for 24 h, and then the thymol from the dried powder of the release medium was extracted with 5 mL of hexane. The concentration of thymol was determined using a UV-VIS spectrophotometer.

### 3.8. Statistical Analyses

All of the results were presented as the mean ± standard deviation (SD) of at least triplicate determinations. The data were analyzed using one-way analysis of variance (ANOVA) with Tukey’s multiple-comparison test to compare the significance among the samples. The significant level (*p*) was set as 0.05.

## 4. Conclusions

In conclusion, the results of this proof-of-concept study suggest that the chitosan hydrogel beads can be successfully functionalized with nanoparticles made from food-derived nanomaterials in order to form multifunctional nanoparticles–hydrogel nanocomposites. The surface-active OD on the SLPN not only stabilized the solid lipid nanoparticles, but also enabled the surface of the nanoparticles to react with the chitosan via Schiff base linkage, forming a covalent conjugation. The formulation and preparation parameters during the conjugation process between SLPN and the chitosan beads, including the beads dosage and incubation duration, were comprehensively investigated. Our results concluded that a 200 beads dosage with a 30–90 min incubation duration was found to achieve a high conjugation efficiency (30–60%) with unaffected physicochemical characteristics of SLPN after conjugation. After the optimization, the thymol was pre-loaded into the SLPN to prepare the antimicrobial nano-on-beads composites that exhibited a sustained release of thymol and thus held great potential for food safety-related applications.

## Figures and Tables

**Table 1:** Particulate characteristics of thymol loaded solid lipid–polymer hybrid nanoparticles (SLPN).

Group	Empty	T-SLPN1	T-SLPN2	T-SLPN3
Thymol loading (*v*/*v*)	0	0.05	0.075	0.1
Particle size (nm)	136.0 ± 5.6	141.1 ± 8.4	140.0 ± 5.6	144.1 ± 3.0
PDI	0.238 ± 0.011	0.241 ± 0.025	0.246 ± 0.032	0.275 ± 0.028
Zeta potential (mV)	−14.2 ± 1.8	−14.3 ± 1.6	−14.4 ± 0.9	−16.3 ± 2.4
EE (%)	NA	93.2 ± 1.6^A^	91.1 ± 1.9^A^	78.3 ± 8.3^B^

The capital superscript letter indicates the significant difference in that parameter among different samples at *p* < 0.05. EE—encapsulation efficiency; PDI—polydispersity index.

**Table 2:** Determination of the coefficient (R^2^) of the fitted model equations applied to the thymol release kinetics.

Group	Zero-Order Model		Higuchi Model		Korsmeyer–Peppas Model	
	Equation	R^2^	Equation	R^2^	Equation	R^2^
200B-30m	y = 0.14x	0.927	y = 2.20x − 1.19	0.945	y = 0.67x − 0.06	0.921
200B-60m	y = 0.16x	0.920	y = 2.44x + 1.18	0.982	y = 0.45x + 0.51	0.974
200B-90m	y = 0.18x	0.914	y = 2.65x + 2.19	0.984	y = 0.45x + 0.57	0.977
